# Muscle Mass and Inflammation in Older Adults: Impact of the Metabolic Syndrome

**DOI:** 10.1159/000520096

**Published:** 2022-01-31

**Authors:** Nikolaus Buchmann, Jens Fielitz, Dominik Spira, Maximilian König, Kristina Norman, Graham Pawelec, David Goldeck, Ilja Demuth, Elisabeth Steinhagen-Thiessen

**Affiliations:** ^a^Department of Endocrinology and Metabolic Diseases (including Division of Lipid Metabolism), Charité − Universitätsmedizin Berlin, Corporate Member of Freie Universität Berlin and Humboldt-Universität zu Berlin, Berlin, Germany; ^b^Department of Internal Medicine B, Cardiology, University Medicine Greifswald, Greifswald, Germany; ^c^Department of Cardiology, Charité − University Medicine Berlin (Campus Benjamin Franklin), Berlin, Germany; ^d^DZHK (German Centre for Cardiovascular Research), Partner site Greifswald, Greifswald, Germany; ^e^Department of Endocrinology and Metabolism, Charité − Universitätsmedizin Berlin, Corporate Member of Freie Universität Berlin, Humboldt-Universität zu Berlin, and Berlin Institute of Health, Berlin, Germany; ^f^Division of Nephrology and Internal Intensive Care, Department of Internal Medicine, Charité − Universitätsmedizin Berlin, Berlin, Germany; ^g^German Institute of Human Nutrition Potsdam Rehbrücke, Department of Nutrition and Gerontology, Nuthetal, Germany; ^h^Institute of Nutritional Science, University of Potsdam, Nuthetal, Germany; ^i^Department of Immunology, University of Tübingen, Tübingen, Germany; ^j^Health Sciences North Research Institute, Sudbury, Ontario, Canada; ^k^Fairfax Centre, Kidlington, United Kingdom; ^l^Berlin Institute of Health Center for Regenerative Therapies, BCRT, Charité − Universitätsmedizin Berlin, Berlin, Germany

**Keywords:** Metabolic syndrome, Muscle mass, Inflammation

## Abstract

**Background:**

Inflammatory processes are a cause of accelerated loss of muscle mass. Metabolic syndrome (MetS) is a highly prevalent age-related condition, which may promote and be promoted by inflammation. However, whether inflammation in MetS (metaflammation) is associated with lower muscle mass is still unclear.

**Methods:**

Complete cross-sectional data on body composition, MetS, and the inflammatory markers interleukin (IL)-1β, IL-6, IL-10, tumor necrosis factor (TNF), and C-reactive protein (CRP) were available for 1,377 BASE-II participants (51.1% women; 68 ± 4 years old). Appendicular lean mass (ALM) was assessed by dual-energy X-ray absorptiometry. Low muscle mass (low ALM-to-BMI ratio [ALMBMI]) was defined according to the Foundation for the National Institutes of Health (FNIH) Sarcopenia Project. Regression models, adjusted for an increasing number of confounders (sex, age, physical activity, morbidities, diabetes mellitus type II, TSH, albumin, HbA1c, smoking habits, alcohol intake, education, and energy intake/day), were used to calculate the association between low ALMBMI and high inflammation (tertile 3) according to MetS.

**Results:**

MetS was present in 36.2% of the study population, and 9% had low ALMBMI. In the whole study population, high CRP (odds ratio [OR]: 2.7 [95% CI: 1.6–4.7; *p* = 0.001]) and high IL-6 (OR: 2.1 [95% CI: 1.2–1.9; *p* = 0.005]) were associated with low ALMBMI. In contrast, no significant association was found between TNF, IL-10, or IL-1β with low ALMBMI. When participants were stratified by MetS, results for IL-6 remained significant only in participants with MetS.

**Conclusions:**

Among BASE-II participants, low ALMBMI was associated with inflammation. Low-grade inflammation triggered by disease state, especially in the context of MetS, might favor loss of muscle mass, so a better control of MetS might help to prevent sarcopenia. Intervention studies to test whether strategies to prevent MetS might also prevent loss of muscle mass seem to be promising.

## Introduction

The term inflamm-aging refers to the increased secretion of proinflammatory cytokines in the old [1]. Immunosenescence, the aging of the immune system, is associated with low-grade systemic and chronic (= subclinical) inflammation [2]. Although acute inflammation is essential for host defense against pathogens, chronic inflammation appears to be harmful in advanced age [3]. Aging alters immune responses in various ways. Not only the decline of immune-active tissue such as the thymus but also a general reduction in the activity of bone marrow, a reduction in functional natural killer cells, less cytokine production by macrophages, or less antibody production are important [4]. This contributes to lower effectiveness of vaccines and a higher susceptibility to infections in old age [2, 4]. In younger subjects, a clear sex-specific difference is evident. While women seem to be less susceptible to infections, they suffer a higher rate of autoimmune disease [5, 6]. Post-menopause, the immune system seems to become more similar in men and women [5, 6]. An increase in serum levels of IL-6, tumor necrosis factor (TNF)-α, and C-reactive protein (CRP) can generally be observed in older adults and the susceptibility to infection and the prevalence of autoimmune disease converge [4]. Nevertheless, there remains a need for research in order to a directly compare the aging immune system in older men and women. This is of general interest, as inflammation contributes to age-associated diseases such as dementia, arteriosclerosis, diabetes mellitus, and also to frailty and sarcopenia (online suppl. Fig. 1; for all online suppl. material, see www.karger.com/doi/10.1159/000520096) [7, 8, 9, 10, 11].

An important transition in aging is the progressive reduction of muscle mass and increase in visceral fat mass [12, 13]. Similar to the aging of the immune system, this is often considered part of the normal aging process. In some cases, however, loss of muscle mass and muscle function result in disease and sarcopenia [12]. Although an International Classification of Diseases (ICD) code for sarcopenia has been established in the USA, there is still no uniform definition of this condition [14, 15]. In addition to the first description proposed by Rosenberg [16] in 1988, in the meantime definitions of the European Working Group on Sarcopenia in Older People (EWGSOP) and the Foundation for the National Institutes of Health (FNIH) Sarcopenia Project have become particularly important [16, 17, 18, 19, 20]. The pathogenesis of muscle wasting is presumed to be multifactorial. Among others, hormonal factors, lifestyle habits such as nutrition and sports activities, and inflammatory processes are involved. Moreover, accelerated loss of muscle mass and muscle function have been observed in the context of age-related diseases [21, 22, 23].

An increase of visceral fat mass is a main feature of the highly prevalent age-related metabolic syndrome (MetS), a cluster of cardiometabolic risk factors that may act as promoters of inflammation (metaflammation) in the absence of infection [24]. The development of MetS should not be viewed as the “normal” course of aging, but nevertheless its prevalence increases with age leading to significant health and economic consequences [25]. We and others have previously described complex relationships between MetS and muscle mass or sarcopenia [22, 26]. Meanwhile, there is increasing evidence of an association between MetS and sarcopenia [26]. However, the role of inflamm-aging/metaflammation in this context has not been extensively investigated thus far [26, 27]. Nevertheless, metaflammation has been reported in the context of MetS and is discussed as a potential proxy for loss of muscle mass and consequent sarcopenia. The aim of the current study was to investigate the relationship of MetS-associated inflammation/metaflammation and muscle mass. To do so, we assessed the relationship between different inflammation parameters (IL-1 β, IL-6, IL-10, TNF, and CRP) and low lean mass in 1,377 participants of the Berlin Aging Study II (BASE-II) stratified by the presence or absence of MetS.

## Methods

### Study Sample

We analyzed data of BASE-II, a convenience sample of relatively healthy, community-dwelling participants, which has been described before [28, 29]. Complete cross-sectional data on body composition, MetS, a number of cytokines, and CRP were available for 1,503 BASE-II participants aged 60 years and older. We excluded participants who reported infectious disease during or up to 1 week prior to the examination (*n* = 97) and participants with missing information on infectious states (*n* = 20). Participants with leukocyte concentrations >11 g/L (*n* = 9) were also excluded, resulting in a sample of 1,377 BASE-II participants analyzed here.

### Body Composition and Definition of MetS and Association with Low ALM

Body composition was assessed with dual-energy X-ray absorptiometry ([DEXA], Hologic QDR Discovery; Hologic Inc., Bedford, MA, USA) with a trained technician performing the DEXA measurement protocol. Appendicular lean mass (ALM) in kilograms was calculated as the sum of the regional lean mass of the 4 limbs. From this, the ALM-to-BMI ratio (ALMBMI) was derived. Cutoff values for low ALMBMI of <0.789 in men and <0.512 in women were set according to the lean mass thresholds for higher likelihood of weakness as identified within the FNIH Sarcopenia Project [19]. Subjects with an ALMBMI below these cutoffs were classified as having low ALMBMI. MetS was defined as suggested by the International Diabetes Federation/American Heart Association/National Heart, Lung and Blood Institute (IDF/AHA/NHLBI 2009) [24].

### Measurement of Parameters of MetS and Laboratory Measurements

Blood pressure was measured with an electronic sphygmomanometer (boso-medicus memory, Jung Willingen, Germany), waist circumference was assessed using a nonelastic tape measure at the level of the umbilicus, and elevated waist circumference was classified as ≥94 cm in men and ≥80 cm in women [24]. Triglycerides and high-density lipoprotein (HDL) cholesterol were measured with enzymatic color tests (Roche/Hitachi Modular; device: ACN 435 und ACN 781). Measurement of glucose level (pre- and post-load) was carried out by photometric concentration determination, insulin levels were analyzed by chemiluminescence immunoassays, and HbA1c was analyzed by ion-exchange high-performance liquid chromatography. An oral glucose tolerance test (OGTT) [30] was performed in BASE-II in participants without previously known diabetes.

### Inflammation

Plasma CRP level was determined using an immunoturbidimetric assay (cobas/Roche, Rotkreuz, Switzerland). Concentrations of serum cytokines IL-1β, IL-6, IL-10, and TNF were measured with the high-sensitivity CBA flex system (BD biosciences) following the manufacturer's instructions but with 1 additional dilution of the standard. To increase the accuracy of the curve, standards were analyzed 3 times. Measurements were performed on a BD LSR-II flow cytometer and analysis carried over with BD templates. To control for constant cytometer performance over the study time, BD CS&T beads were employed.

### Confounders

To estimate usual nutrient intake, participants completed a validated, self-administered 146-item EPIC-FFQ Potsdam Germany (European Prospective Investigation into Cancer and Nutrition) [31, 32, 33]. Regular alcohol consumption (yes/no) and current smoking status (yes/no) were assessed by a standardized questionnaire. As part of the medical examination, diagnoses were obtained through participant reports, with selected diagnosis (e.g., diabetes mellitus) being verified by additional blood laboratory tests. Diagnoses were used to compute a morbidity index largely based on the categories of the Charlson Comorbidity Index, which is a weighted sum of moderate to severe, mostly chronic physical illnesses, including cardiovascular (e.g., congestive heart failure), cancer (e.g., lymphoma), and metabolic diseases (e.g., diabetes mellitus) [34, 35]. We used the Rapid Assessment of Physical Activity (RAPA) questionnaire to assess physical activity of the study population [36].

### Statistics

**Fig. 1:**
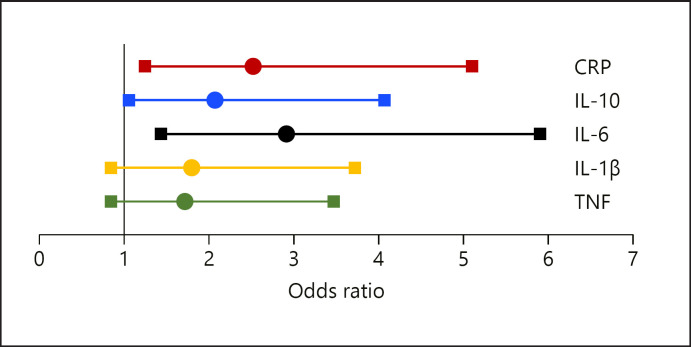
ORs for low lean mass in subjects without MetS according to inflammation markers. CRP, C-reactive protein; IL, interleukin; TNF, tumor necrosis factor; MetS, metabolic syndrome; OR, odds ratio.

Statistical analyses were carried out using the software package IBM^ Statistics SPSS 25. Data are given in percentages or as medians and interquartile range as indicated. The Mann-Whitney U-test was performed to compare means between groups. The χ^2^ test was used to compare proportions between groups. Cytokine concentrations and CRP were divided into tertiles for further calculations. In adjusted regression models, we estimated the association between low lean mass (ALMBMI below cutoff) as the dependent variable and “high cytokine levels” (tertile 3 vs. tertiles 1 + 2) as explanatory variables. The same models were calculated for the total study population, and separately for subjects without MetS and for subjects with MetS. Additionally, for each different cytokine studied, separate models were calculated. We adjusted for an increasing number of potential confounders with model 3 being the fully adjusted model, adjusted for sex, age, morbidities, diabetes, physical activity, thyroid-stimulating hormone (TSH), albumin, HbA1c, smoking habits, alcohol intake, years of professional education, and total energy intake. Figure 1 and online supplementary Figures 1–3 display odds ratios (ORs) resulting from the regression models (model 3). To rule out that 2 or more of the variables correlate strongly with each other, multicollinearity tests were performed and VIF values above 5 were considered “too high.” Effect size was measured according Cohen's f statistic [37]. Considering multiple comparisons, an acceptable level of statistical significance was established a priori at *p* < 0.01 (significance level adjusted according to Bonferroni).

## Results

### General Characteristics of the Study Population

1,377 BASE-II participants were analyzed in this study (51.1% women; 68 ± 4 years old). MetS was present in 36.2%, and 9% had low ALMBMI. These characteristics and details regarding CRP and cytokine concentrations are summarized in online supplementary Table 1 according to the ALMBMI cutoff. Participants with low ALMBMI were older, had a higher morbidity score, and had higher concentrations of CRP and IL-6. The levels of the other cytokines analyzed here − IL-1β, IL-10, and TNF − were comparable in those with and without low ALMBMI. MetS was more common in subjects with ALMBMI below the cutoff, relative to participants with ALMBMI above the cutoff, and more women exhibited low ALMBMI. In addition, physical inactivity was reported more frequently in participants with low ALMBMI.

Characteristics of the participants with low ALMBMI and MetS are given in Table 1. *p* values and other parameters were separately computed for subjects with and without MetS according to low ALMBMI. With respect to constellations of markers of inflammation, participants with MetS and low ALMBMI had higher concentrations of CRP, IL-6, and IL-10 than subjects with MetS but ALMBMI above the cutoff. Participants without MetS and with low ALMBMI also had higher concentrations of CRP and IL-6, but not IL-10 compared to subjects without MetS and with ALMBMI above the cutoff.

### Model Results

Results of the regression models focusing on the association between CRP and low ALMBMI are shown in Table 2. Models 1–3 were analogously computed for IL-6 (Table 3), IL-10 (online suppl. Table 2), TNF (Fig. 1, online suppl. Fig. 2, 3), and IL-1β (Fig. 1, online suppl. Fig. 2, 3). As the results did not change significantly according to adjustment, the following results refer to the fully adjusted model 3, and Figure 1 and online supplementary Figures 2 and 3 display the results of regression model 3.

Regarding CRP and IL-6, we found a positive association with low lean mass in the whole study population. The OR for low ALMBMI was 2.7 (95% CI: 1.6–4.7; *p* = 0.001) comparing participants with high CRP concentrations (tertile 3; concentrations above 1.5 mg/dL) to those with low concentrations (tertiles 1 and 2) and the OR for low ALMBMI was 2.1 (95% CI: 1.2–1.9; *p* = 0.005) for participants with high IL-6 concentrations (tertile 3; concentrations above 2.4 pg/mL) compared to subjects with lower IL-6 concentrations, respectively.

Importantly, when we stratified for MetS and performed the same calculations, we found that the association between low ALMBMI and high IL-6 concentrations was only robust in participants with MetS (OR 2.9 [95% CI: 1.4–5.9; *p* = 0.003]) but not for participants without MetS. In addition, we observed no association between IL-10 and low ALMBMI in the whole sample. Moreover, no association between high IL-10 concentrations and low ALMBMI was found in subjects with or without MetS. Specifically, the OR was 2.1 (95% CI: 1.1–4.1; *p* = 0.033) for participants with MetS if they had high IL-10 concentrations (tertile 3; concentrations above 0.49 pg/mL), however, due to multiple comparisons, this result remained insignificant applying Bonferroni correction.

We recalculated the highest adjusted model with respect to the IL-6-low ALMBMI association including high (tertile 3) concentrations of IL-10 (model 4) and low (tertile 1) IL-10 concentrations (model 5); however, results remained stable. We found no significant association between TNF and IL-1β with low ALMBMI.

## Discussion

The current cross-sectional analysis of 1,377 BASE-II participants focused on associations between inflammatory patterns and low lean mass defined by the FNIH approach. Moreover, the aim of the current study was to investigate the relationship of MetS-associated inflammation/metaflammation and muscle mass. We found that the proinflammatory cytokine IL-6 and CRP concentrations were higher in subjects with low ALMBMI. We were particularly interested if there is an effect modification by the presence of MetS, and therefore stratified our analysis according to MetS. In stepwise adjusted regression models, we found that high concentrations of IL-6 were associated with increased odds of low ALMBMI only in subjects with MetS, whereas high CRP concentrations were associated with low ALM independent of MetS.

### Inflammation and Muscle Mass

Inflammatory processes are major factors for the development of low lean mass or sarcopenia, respectively [9, 38, 39]. Recently, we have shown that IL-1β is associated with muscle atrophy in a mouse model of polymicrobial sepsis [40]. In addition, elevated serum levels of IL-6 were associated with muscle failure of critically ill patients [41]. However, it is important to differentiate between acute and chronic effects of proinflammatory cytokines. For example, on the one hand IL-6 is associated with muscle growth and myogenesis and is released by muscle cells during exercise [42], but on the other hand, IL-6 and other proinflammatory cytokines have been shown to accelerate muscle wasting and atrophy in chronic inflammation [43]. Thus, it is suggested that chronic inflammation may be associated with a decline of physical functioning through the catabolic effects of inflammation on muscle (online suppl. Fig. 1) [44]. In a recent meta-analysis, Bano et al. [38] reported that sarcopenia was associated with higher serum CRP, but not with higher IL-6 or TNF concentrations compared to nonsarcopenic subjects. Contrary to these findings, Thomas [45] concluded in their review that sarcopenia may result from other mechanisms such as hormonal and neurodegenerative changes in age, but not necessarily inflammation. Our results from BASE-II are mainly in line with the latter findings. We show that CRP was higher in participants with low ALMBMI, independent of MetS. However, in subjects with low ALMBMI but without MetS, higher levels of IL-6 and IL-10 or alterations in TNF were not observed.

### Inflammation and MetS

MetS is characterized by central obesity with dyslipidemia, elevated blood pressure, and insulin resistance, and is associated with worse outcomes [24, 46]. Secretory products of adipose tissue include (adipo-)cytokines such as TNF, IL-6, and adiponectin, which are responsible for the crosstalk between adipose tissue and other target organs. Increased (visceral) fat tissue drives the formation of messengers (i.e., leptin, adiponectin, and plasminogen activator inhibitor-1) and the inflammatory molecules TNF, IL-6, and CRP [47]. IL-6 is a main driver in body energy homeostasis (e.g., regulation of appetite, inhibition of lipoprotein-lipase activity) (see online suppl. Fig. 1). Adiponectin has a wide range of effects on lipid and glucose metabolism, and in particular, it increases the sensitivity of target tissues to insulin and plays a major role in the context of MetS [48].

Moreover, TNF and IL-6 are involved in lipid metabolism and in insulin signaling [49]. TNF and IL-6 have also been shown to downregulate anti-inflammatory cytokines such as adiponectin [50]. The dyslipidemia found in MetS subjects may be associated with low lean mass through adiponectin-mediated mechanisms. However, an association between low HDL-C and elevated triglycerides and inflammation with low lean mass might additionally be affected by lifestyle behaviors, such as sports and nutrition, which can affect lipid concentrations and inflammatory patterns. The current analysis supports this hypothesis because subjects with MetS are likely to be physically inactive to a greater extent. Although total energy intake did not differ significantly between participants with and without MetS, dietary habits might play an additional role, which could not be analyzed in detail here.

### Inflammation, Muscle Mass, and MetS

Associations between inflammation markers and loss of muscle mass and muscle strength have been published before, and the role of obesity in this context has already been addressed [38, 45, 51, 52, 53]. An association between obesity, inflammation/metaflammation, and sarcopenia has been particularly well-studied in the context of sarcopenic obesity (SO) and associations between MetS and SO have also been reported [52, 54]. Elevated concentrations of IL-6 and CRP are often detected in sarcopenia and SO. Schrager et al. [52] concluded in an analysis of the InCHIANTI study that central obesity affects inflammation and may therefore impair muscle function and promote SO. Sente et al. [55] investigated the effects of proinflammatory cytokines on cultured skeletal muscle cells isolated from heart failure patients. Their results suggest that proinflammatory cytokines lead to loss of muscle mass and muscle function through a FOXO3a-mediated pathway and impaired adiponectin signaling [55]. The latter may lead to loss of muscle mass and muscle function. Moreover, low lean mass defined by the FNIH approach, as we did in the current analysis, has already been linked to inflammation in a large analysis of National Health and Nutrition Examination Survey (NHANES) data (increased concentrations of fibrinogen and CRP in SO-participants) [56]. However, the authors focused on sarcopenia and SO in an older population and not all cytokines studied in the current analysis were measured in NHANES. Notably, using the FNIH approach, subjects with low lean mass often also exhibit higher fat mass due to the ALM-to-BMI ratio used. The same is true for subjects with MetS as increased visceral fat mass is a hallmark of MetS. We observed an association between low ALMBMI with CRP, the proinflammatory cytokine IL-6, but not with the anti-inflammatory cytokine IL-10 (which did not achieve statistical significance after correction for multiple testing). We found no associations with other cytokines such as TNF or IL-1β. Assuming the balance of pro-/anti-inflammatory cytokines could have influenced the results, we additionally calculated regression models including both high IL-6 concentrations and high (Table 3; model 4)/low (Table 3; model 5) IL-10 concentrations. However, these results remained unchanged. Upregulation of anti-inflammatory cytokines, possibly compensatory in the context of systemic inflammation, has previously been described, and metaflammation is particularly pronounced in MetS [57, 58]. Thus, it seems plausible that the relationship between IL-10 and muscle mass could be found in MetS, but due to multiple testing in our analysis and a relatively small study sample, we could not document a significant association. Another factor in this context is the group of subjects examined here was relatively healthy. It might be assumed that results differ in more multimorbid study populations.

A relationship between TNF signaling and muscle protein breakdown has been studied in animal models, but translation to humans has not been uniform [59, 60]. With respect to muscle mass, it has been shown that several cytokines are upregulated during and after exercise. An increase of TNF and IL-1β was accompanied by an even more marked increase in IL-6 [61]. Indeed, MetS is associated with increased TNF, IL-6, and free fatty acid (FFA) levels, which activate proteolysis in skeletal muscle. In obese individuals, insulin resistance promotes muscle catabolism because insulin is a powerful anabolic signal [62]. TNF may well be found in sarcopenia and MetS, but is not as pronounced as found in the current study. Notably, also in our analysis, for example, TNF levels were higher in participants with MetS and low ALMBMI, and the highest levels of TNF were seen in subjects with MetS and low ALMBMI, but these differences did not reach statistical significance.

### Limitations

The current study is subject to certain limitations. Given the cross-sectional design of the BASE-II dataset, conclusions regarding causalities cannot be drawn. Moreover, BASE-II is a convenience sample, and the participants are on average healthier than the general population [63, 64]. Nevertheless, MetS was frequently present in this population, as was low lean mass. Regular alcohol intake, total energy intake, current smoking status, and physical activity were assessed by standardized questions. However, under- or over-reporting is possible even when using validated questionnaires. With respect to the statistical analyses, although we adjusted for a considerable number of confounding factors, we cannot exclude the possibility that the results may be affected by, for example, further dietary factors or medication use that we could not consider. For the current analysis, we decided to use the approach suggested by FNIH, because the subjects included in this approach to create cutoff points for low muscle mass have certain similarities to the BASE-II participants. However, we focused on low lean mass, and there is still a need for further research to clarify associations between sarcopenia and metaflammation/inflammation.

## Conclusions and Outlook

In conclusion, in the current analysis of BASE-II data, participants with MetS had higher indicators of inflammation and these (CRP and IL-6) were associated with low lean mass. We hypothesize that metabolic alterations and the accompanying low-grade inflammation (metaflammation) accelerate the loss of muscle mass by hormonal, metabolic, and inflammatory processes. Low-grade inflammation might contribute to disease states such as insulin resistance, dyslipidemia, and obesity. Thus, low-grade inflammation triggered by MetS (metaflammation) might favor loss of muscle mass, whereas low-grade inflammation occurring during normal aging (inflamm-aging) might not be harmful enough to see such effects on muscle. In clinical practice, it seems to be important to identify subjects with metabolic alterations, especially MetS, as they may be at increased risk for muscle mass decline and associated disease. In general, the treatment of underlying diseases that promote inflammation/metaflammation could be useful to prevent age-dependent decline of muscle mass, thus increasing dependence. The detection of MetS is possible through simple and inexpensive laboratory and anthropometric measurements. Thus, screening subjects with MetS for sarcopenia might serve as a cost-effective tool to prevent dependency at advanced age. As nutritional interventions and physical activity are the most promising interventions to date for the prevention of sarcopenia, especially in subjects with MetS, this should be addressed to prevent early loss of muscle mass and physical dependency in old people.

Nevertheless, there is still need for further research in this field. Although an association between inflammation and muscle mass is well-documented, the influence of metabolic disease in this context needs further exploration. Especially, hormonal issues, such as changes in metabolically active hormones (at menopause) and sex differences, should be addressed to generate further insights into the complex interplay between muscle, inflammation, and metaflammation. Including factors for muscle function (i.e., EWGSOP2 definition criteria for sarcopenia) might result in additional insights with respect to sarcopenia.

## Statement of Ethics

All subjects gave written informed consent to participate in the study. The study was conducted according to the Declaration of Helsinki and was approved by the Ethics Committee of Charité − University Medicine Berlin (Project Number: EA2/029/09).

## Conflict of Interest Statement

The authors do not report any financial or personal conflicts of interest related to the content of this research. They do not have any financial or non-financial interests which affected the manuscript.

## Funding Sources

The BASE-II research project (Co-PIs are Lars Bertram, Ilja Demuth, Denis Gerstorf, Ulman Lindenberger, Graham Pawelec, Elisabeth Steinhagen-Thiessen, and Gert G. Wagner) was supported by the German Federal Ministry of Education and Research (Bundesministerium für Bildung und Forschung, BMBF) under Grant Numbers 01UW0808, 16SV5536K, 16SV5537, 16SV5538, 16SV5837; 01GL1716A, and 01GL1716B), and by the Max Planck Institute for Human Development, Berlin, Germany. Additional contributions (e.g., equipment, logistics, personnel) are made from each of the other participating sites. Further details about the study can be found at https://www.base2.mpg.de/en.

## Author Contributions

Substantial contributions to the conception or design of the work: Dr. med. Nikolaus Buchmann, Prof. Dr. med. Jens Fielitz, Prof. Dr. Ilja Demuth, and Prof. Dr. med. Elisabeth Steinhagen-Thiessen. Acquisition, analysis, or interpretation of data for the work: Dr. med. Nikolaus Buchmann, Dr. med. Dominik Spira, Dr. med. Maximilian König, Prof. Dr. med. Kristina Norman, Dr. Graham Pawelec, and Dr. David Goldeck. Drafting the work or revising it critically for important intellectual content: Dr. med. Nikolaus Buchmann, Prof. Dr. med. Jens Fielitz, Dr. med. Dominik Spira, Dr. med. Maximilian König, Prof. Dr. med. Kristina Norman, Prof. Dr. Graham Pawelec, Dr. David Goldeck, Prof. Dr. Ilja Demuth, and Prof. Dr. med. Elisabeth Steinhagen-Thiessen. Final approval of the version to be published: Dr. med. Nikolaus Buchmann, Prof. Dr. med. Jens Fielitz, Dr. med. Dominik Spira, Dr. med. Maximilian König, Prof. Dr. rer. medic. Kristina Norman, Prof. Dr. Graham Pawelec, Dr. David Goldeck, Prof. Dr. Ilja Demuth, and Prof. Dr. med. Elisabeth Steinhagen-Thiessen. Agreement to be accountable for all aspects of the work in ensuring that questions related to the accuracy or integrity of any part of the work are appropriately investigated and resolved: Dr. med. Nikolaus Buchmann, Prof. Dr. med. Jens Fielitz, Dr. med. Dominik Spira, Dr. med. Maximilian König, Prof. Dr. rer. medic. Kristina Norman, Prof. Dr. Graham Pawelec, Dr. David Goldeck, Prof. Dr. Ilja Demuth, and Prof. Dr. med. Elisabeth Steinhagen-Thiessen.

## Data Availability Statement

Due to concerns for participant privacy, data are available only upon request. External scientists may apply to the Steering Committee of BASE-II for data access. Please contact Katrin Schaar, scientific coordinator, at schaar@mpib-berlin.mpg.de.

## Supplementary Material

Supplementary data

Supplementary data

Supplementary data

## Figures and Tables

**Table 1:** Characteristics of the study population according to low ALMBMI and MetS

	ALMBMI > cutoff		*p* value	ALMBMI < cutoff		*p* value
	no MetS	MetS		no MetS	MetS	
Female, *n* (%)	475 (57.0)	184 (43.8)	ns	17 (37.0)	28 (35.9)	0.006
Age, years	68.6 (65.8–71)	68.4 (65.3–719)	0.026	69.7 (67.2–73.2)	69.5 (66.9–71.7)	0.017
BMI, kg/m^2^	24.9 (23.01–27.1)	27.81 (26.06–30.23)	<0.001	29.07 (27.33–31.35)	32.85 (29.73–37.48)	<0.001
ALM, g	19.5 (16.4–24.3)	23.6 (18.2–26.8)	0.011	20.0 (15.1–22.5)	20.8 (16.6–24.6)	ns
Professional education, years	4 (3–5)	4 (3–5)	ns	3.5 (3–5)	3.5 (3–5)	ns
Total energy/day, kcal/day	2,081 (1,721–2,573)	2,174 (1,770–2,685)	ns	2,398 (1,796–2,766)	2,271 (1,754–2,686)	ns
Physical inactivity, *n* (%)	49 (5.9)	56 (13.4)	0.046	7 (16.7)	16 (21.1)	0.018
Current smoking, *n* (%)	66 (9.4)	32 (9.1)	ns	4 (11.4)	7 (11.1)	ns
Regular alcohol intake, *n* (%)	629 (89.3)	327 (92.9)	ns	32 (91.4)	54 (85.7)	ns
CRP, mg/L	1 (0.5–1.7)	1.3 (0.7–2.6)	<0.001	1.7 (1–2.7)	2.6 (1.4–4.6)	<0.001
Circulating TNF, pg/mL	0.18 (0–0.53)	0.10 (0–0.42)	ns	0.15 (0–0.45)	0.21 (0–0.44)	ns
Circulating IL-6, pg/mL	1.77 (1.07–2.74)	1.93 (1.36–3.10)	<0.001	2.22 (1.50–3.59)	3.22 (1.87–4.33)	0.006
Circulating IL-1β, pg/mL	0.081 (0–0.391)	0.019 (0–0.263)	ns	0.026 (0–0.247)	0.033 (0–0.286)	ns
Circulating IL-10, pg/mL	0.37 (0.17–0.67)	0.35 (0.18–0.59)	0.035	0.41 (0.27–0.57)	0.41 (0.27–0.81)	ns

As cutoff values for low ALMBMI, <0.789 in men and <0.512 in women were chosen according to the lean mass thresholds for higher likelihood of weakness as identified within the FNIH Sarcopenia Project. Subjects with an ALMBMI below these cutoffs were classified as participants with low ALMBMI. BMI, body mass index; ALM, appendicular lean mass; CRP, C-reactive protein; IL, interleukin; TNF, tumor necrosis factor; ALMBMI, ALM-to-BMI ratio; MetS, metabolic syndrome; FNIH, Foundation for the National Institutes of Health.

**Table 2:** Association between low ALMBMI and high CRP concentrations (tertile 3)

CRP	(a) Total population		(b) No MetS		(c) MetS	
	OR (95% CI)	*p* value	OR (95% CI)	*p* value	OR (95% CI)	*p* value
Model 1	2.92 (1.98–4.29)	0.001	2.43 (1.31–4.51)	0.005	3.75 (2.23–6.30)	0.001
Model 2	2.85 (1.85–4.40)	0.001	2.80 (1.33–5.66)	0.004	3.23 (1.83–5.70)	0.001
Model 3	2.74* (1.61–4.68)	0.001	5.00** (1.97–12.57)	0.003	2.52*** (1.25–5.06)	0.010

Model 1: adjusted for sex and age. Model 2: model 1 + physical activity, morbidities, diabetes mellitus type II. Model 3: model 2 + TSH, albumin, HbA1c, smoking, alcohol intake, professional education years, total energy intake/day. ALMBMI, ALM-to-BMI ratio; MetS, metabolic syndrome; TSH, thyroid-stimulating hormone; CRP, C-reactive protein; OR, odds ratio.

* R^2^ = 0.192, Cohens *f* = 0.24.

** R^2^ = 0.229, Cohens *f* = 0.3.

*** R^2^ = 0.180, Cohens *f* = 0.22.

**Table 3:** Association between low ALMBMI and high IL-6 concentrations (tertile 3)

IL-6	(a) Whole population		(b) No MetS		(c) MetS	
	OR (95% CI)	*p* value	OR (95% CI)	*p* value	OR (95% CI)	*p* value
Model 1	2.28 (1.57–3.32)	0.000	1.55 (0.85–2.845)	0.155	3.17 (1.92–5.21)	0.001
Model 2	2.13 (1.40–3.25)	0.001	1.76 (0.89–3.47)	0.105	2.65 (1.53–4.61)	0.001
Model 3	2.14* (1.25–3.66)	0.005	1.57** (0.66–3.77)	0.309	2.91 *** (1.44–5.90)	0.003
Model 4	2.12 (1.24–3.63)	0.006	1.6 (0.67–3.98)	0.028	2.86 (1.41–5.81)	0.004
Model 5	2.18 (1.26–3.78)	0.006	1.58 (0.66–3.81)	0.304	3.167 (1.49–6.75)	0.003

Model 1: adjusted for sex and age. Model 2: model 1 + physical activity, morbidities, diabetes mellitus type II. Model 3: model 2 + TSH, albumin, HbA1c, smoking, alcohol intake, professional education years, total energy intake/day. Model 4: model 3 + high IL-10 concentrations (tertile 3). Model 5: model 3 + low IL-10 concentrations (tertile 1). ALMBMI, ALM-to-BMI ratio; IL, interleukin; MetS, metabolic syndrome; TSH, thyroid-stimulating hormone; OR, odds ratio.

* *R*^2^ = 0.178, Cohens *f* = 0.22.

** R^2^ = 0.169, Cohens *f* = 0.2.

*** R^2^ = 0.190, Cohens *f* = 0.24.

## Notes

Ilja Demuth and Elisabeth Steinhagen-Thiessen shared last authorship.
