# Supplementary figures and images for: Muscle Mass and Inflammation in Older Adults: Impact of the Metabolic Syndrome
Source: Gerontology. 2022 Jan 31;68(9):989–98. doi: 10.1159/000520096 (PMC9501741; doi:10.1159/000520096)

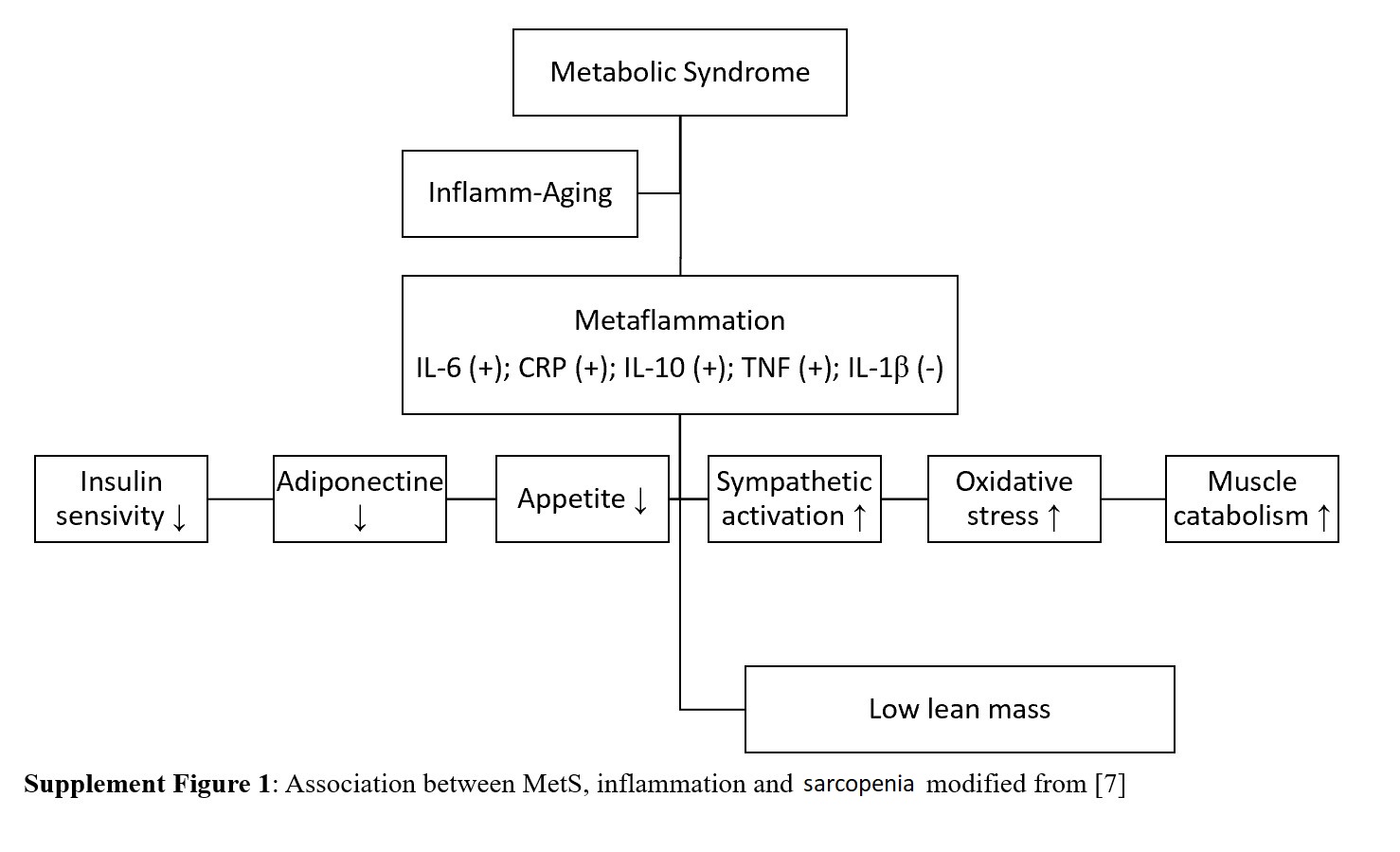

Supplement: Supplementary file 1 — Supplementary data [file ger-0068-0989-s01.jpg]

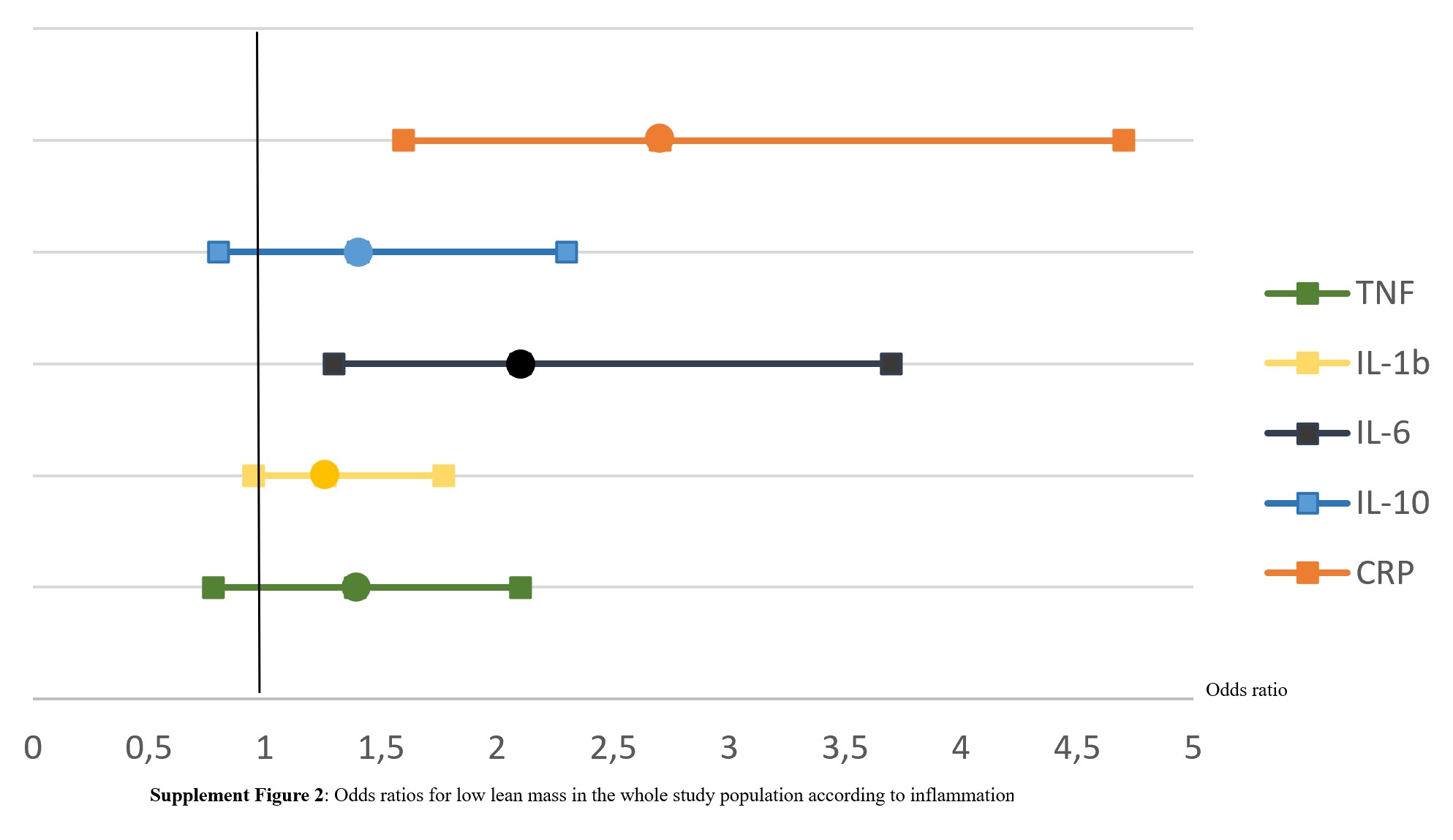

Supplement: Supplementary file 2 — Supplementary data [file ger-0068-0989-s02.jpg]

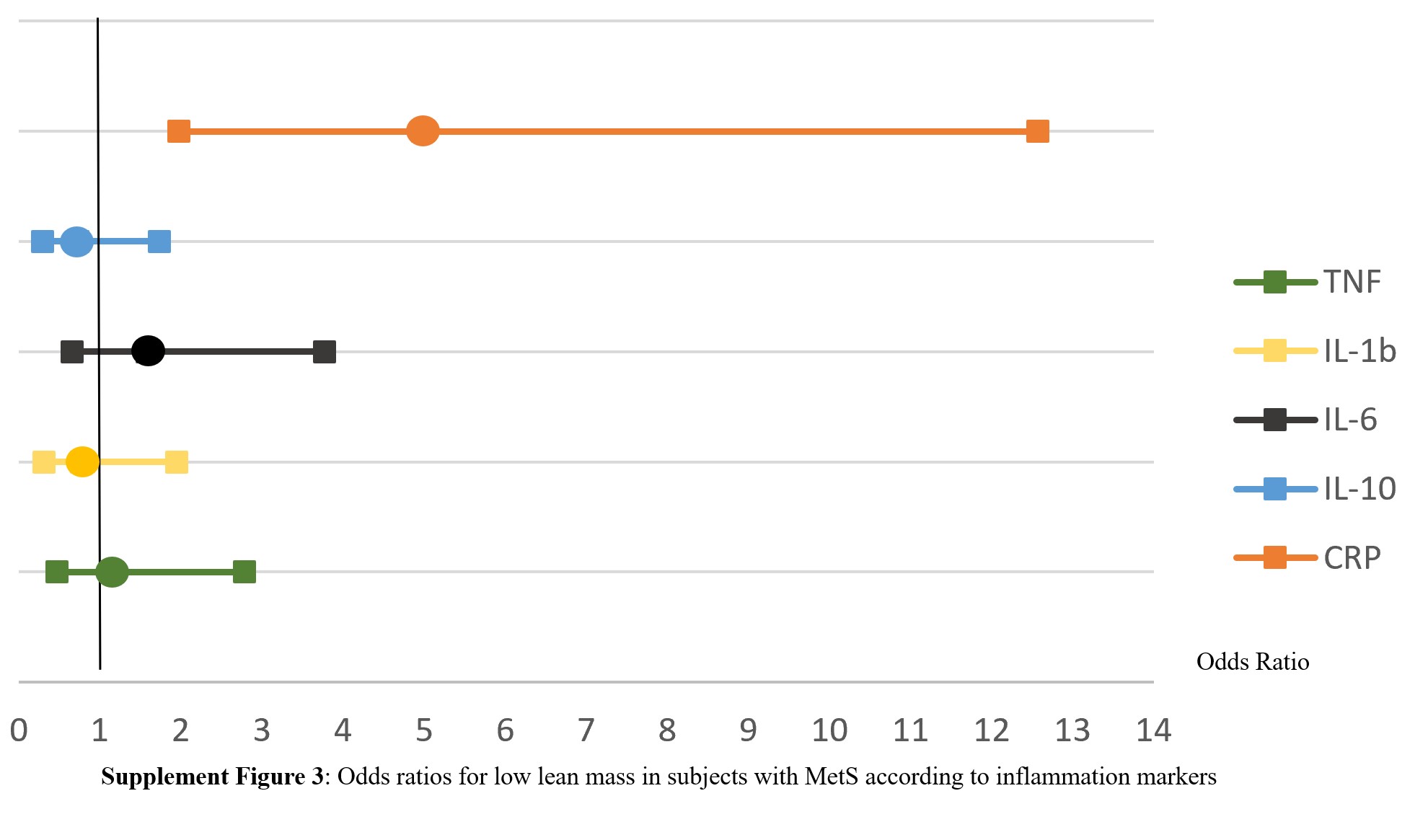

Supplement: Supplementary file 3 — Supplementary data [file ger-0068-0989-s03.jpg]
